# The British Medical Association and the General Medical Council

**Published:** 1906-06-09

**Authors:** 


### The Hospital

A Journal of

The Medical Sciences and Hospital Administration.

Vol. XL.—No. 1,028. SATURDAY, JUNE 9, 1906.

### THE BRITISH MEDICAL ASSOCIATION AND THE GENERAL MEDICAL COUNCIL.

The position and influence of the medical profession in the general community may be said to rest mainly on two factors. In the first place is the importance of the duties which the medical practitioner discharges. Secondly, and of not less weight, there is the general conviction that no consideration will prevent those duties being discharged, in the last resort, in the service of man and not in the service of profit. The medical labourer, like every other labourer, is, of course, worthy of his hire. But the position which he occupies as a contributor to personal and national welfare is not measured by the scale of his fees, but by the knowledge that motives other and higher than these are ever his first consideration. It is that essential and elementary fact which raises medicine to the level of a liberal profession, dignifies it with moral worth, and attracts to its ranks men of large and generous mind. There is yet another influence which acts forcibly in these directions. It is the knowledge that the profession of medicine offers a free atmosphere. There are neither orthodox formulas to be swallowed nor narrow trade rules to be obeyed. The man of honest and candid mind has opportunities for his faculties, and freedom to use these according to the dictates of his own independent judgment. His thought and work may be pursued without fear either of official censure or of organised clamour, and this, even though his conclusions, whether in theory or practice, differ from those adopted by the majority of his fellows.

The preservation of the positions so defined is an interest of the first moment to the medical profession, as it is upon these that its capacity for influence and guidance over the individual patient and the general public essentially depends. Once permit the suspicion that medicine admits to its counsels narrow and selfish motives, or that professional organisations have the power to restrict and penalise independent opinion, and the larger influences of the profession are at an end. Hence it is a public as well as a professional interest that all who have opportunities for influence shall use these to advocate the maintenance of the liberal and generous traditions of which medicine can boast, and shall protest against any invasion of these either by individual or corporate action.

There have been in recent times certain activities which suggest that influences are arising in the profession alien to the spirit of freedom and independence. One of the most conspicuous of these is the determination of the British Medical Association to nominate candidates for election as direct representatives on the General Medical Council under conditions which exhibit a spirit that can only be described as that of a narrow and intolerant trades unionism. For the moment we say nothing on the general policy of such nominations. But allowing that to pass, it is urgent to direct the attention of the profession to the conditions which the Association will inflict upon those who agree to submit to the compliment of an official nomination. The proposal is that the representative meeting which will assemble next July shall consider the names of those nominated by the several Divisions of the Association, and shall elect two of these for formal commendation to the constituency. But no name will be accepted for discussion unless the holder has first given a signed undertaking (1) that he will not, should he fail to be selected, allow himself to be nominated in opposition to the official candidates; and (2) that if elected to the General Medical Council he will endeavour, as a member of that Council, to give effect to the wishes and opinions of the British Medical Association as these may be declared from time to time. In short, the ambition of the organisers of this scheme is to place on the General Medical Council two delegates, who will be sensitive to the pressure of the Association which they control.

That the general body of the profession approve of this proposal we cannot believe, and this is confirmed by the fact that so far very few of the Divisions have exercised the doubtful privilege thus conferred upon them. But in the presence of active organisation mere indifference means defeat, and we hope that independent members of the profession will speak out in condemnation of this offensive introduction of the methods of the political caucus into medical affairs. It can hardly be credited that men of high character and independent spirit will submit to the test pledge which the Association proposes to administer and will consent to appear in the General Medical Council—a body charged with delicate executive and judicial functions—labelled as delegates under the control of an outside organisation. Nor is it likely that the profession desires its "direct representatives" to appear in this unworthy guise. On the contrary, medical men who value for themselves the right of independent judgment and freedom of speech will not ask a representation inconsistent with this right. Delegates subservient to an imposed policy are not fit representatives of the members of a liberal profession.

